# Medical Students’ Attitudes about Team-Based Learning in a Pre-Clinical Curriculum

**DOI:** 10.3885/meo.2009.Res00280

**Published:** 2009-01-07

**Authors:** Dean X. Parmelee, Dan DeStephen, Nicole J. Borges

**Affiliations:** * Departments of Psychiatry and Pediatrics; † Academic Affairs, Boonshoft School of Medicine Wright State University, Dayton, OH, USA; ‡ Department of Communication; § Center for Teaching and Learning, Wright State University, Dayton, OH, USA; ¶ Department of Community Health, Academic Affairs, Boonshoft School of Medicine Wright State University, Dayton, OH, USA

**Keywords:** attitudes, team-based learning, medical students

## Abstract

**Background:**

Team-Based Learning is relatively new in medical education. Team-Based Learning was integrated into one medical school's pre-clinical curriculum in 2002. Purpose: This study compared how medical students’ attitudes about the Team-Based Learning process changed between the first and second year of medical school.

**Method:**

180 students responded to 19 statements regarding their attitudes about Team-Based Learning during their first and second year of medical school. Data were analyzed using a Mann-Whitney U test. Results: Significant changes in attitudes occurred in the areas of Professional Development, Satisfaction with Team Experience, and Satisfaction with Peer Evaluation but not in the areas of Team Impact on Quality of Learning and Team Impact on Clinical Reasoning Ability.

**Conclusion:**

This study demonstrates that students’ attitudes about working within teams, their sense of professional development, and comfort and satisfaction with peer evaluation change in a curriculum using Team-Based Learning.

Team-Based Learning^1^–^2^ is relatively new in medical education,^3^ although it has been implemented in other educational curricula for years.^4^,^5^ Three modes of instruction are typically present in medical school curricula: Lecture-based, Problem-Based, and a combination of lecture-based with small group teaching. Lecture-Based instruction has been the most common strategy, but it has been challenged over the years because it is a passive form of learning.^6^,^7^ Adding small group teaching to a lecture-based program intends to increase active learning but usually results in more lectures by more faculty. As medical educators recognized the importance of active learning strategies,^8^,^9^ applications of Problem-Based Learning were implemented.^10^ While Problem-Based Learning was introduced years ago^11^–^13^ and has been well studied,^10^,^19^ Team-Based Learning is the newest strategy.^3^

Team-Based Learning is learner-centered but instructor-led, uses a very structured individual and group accountability process, and requires small groups to work together to solve problems.^20^ Team-Based Learning has been described as bringing “together theoretically based and empirically grounded strategies for incorporating the effectiveness of small-group learning into large-group, lecture-oriented sessions”.21 (p.40). There are several essential components to the strategy: 1) *advanced preparation*: the instructor defines what the students must master before coming to class; 2) *team formation*: the instructor assigns students to teams of 5-7 using a transparent process, insuring that all teams have a diversity of backgrounds, experiences, abilities amongst their members; 3) *readiness assurance*: the instructor administers a test composed of multiple choice questions (MCQs) to each student at the start of the class, then all teams take the same test and a group score is generated; 4) *group application exercise*: the instructor has all teams work on a set of very challenging questions, usually in MCQ format; extensive whole class discussion ensues with debate on team choices; 5) *peer evaluation*: students must evaluate each of their team members for his/her contribution to the team's productivity.^22^

Although the application of Team-Based Learning to various courses in medical school^23^–^26^ and health professions education^22^ has been described, a void in the literature exists regarding the impact of Team-Based Learning on medical students’ attitudes. Team-Based Learning was integrated into our medical school's first year and second year curriculum in 2002. During the first two years of a largely lecture-based curriculum, Team-Based Learning sessions were developed in all courses to either supplement or replace lecture material; almost all of the existing small group sessions were replaced with Team-Based Learning. Although the curriculum remained highly dependent upon lectures, the Team-Based Learning sessions provided many active learning sessions in small group format. We felt that it was important to explore students’ attitudes about working within teams given that it was a new teaching approach in our medical school's curriculum. This study compared how medical students’ attitudes about working within teams in Team-Based Learning changed between the first and second year of medical school.

## Method

With institutional review board approval, 180 first-year medical students from the Classes of 2006 (n = 90) and 2007 (n = 90) participated in this longitudinal study. Students from the Class of 2006 completed the questionnaire during their first year of medical school (i.e., 2002) and during their second year of medical school (i.e., 2003), and the Class of 2007 completed the questionnaire during their first year of medical school (i.e., 2003) and during their second year of medical school (i.e., 2004). Response rates were 100% for each class year. In a classroom setting and during class time, students from each class year completed an anonymous questionnaire during their first year of medical school regarding their attitudes about Team-Based Learning. The same questionnaire was given to the students during their second year of medical school. During their first and second year of medical school, the questionnaire was completed by students at the beginning of the year, mid-year, and end of year. Scores from the beginning of the year, mid-year, and end of year were averaged resulting in an overall score for year 1. The same procedure was used to determine the average overall score for year 2.

The questionnaire used in this study consisted of 19 statements with Likert-type responses ranging from Strongly Disagree (1) to Strongly Agree (5). Statements were grouped using 5 categories: Overall Satisfaction with Team Experience, Team Impact on Quality of Learning, Satisfaction with Peer Evaluation, Team Impact on Clinical Reasoning Ability, and Professional Development. The questionnaire used in this study was based on the Minnesota Satisfaction Questionnaire.^27^ The specific questions on the survey were developed over a five year timeline within Wright State University's Department of Communication's Organizational Communication classes. Specific questions on the survey can be found in a variety of studies on participants’ satisfaction with their group experiences.^28^–^32^

Of the 180 participants, 95 (53%) were female and 85 (47%) were male. With regard to ethnicity, there were 156 (87%) Caucasians, 20 (11%) African Americans, 3 (1.5%) Mexican Americans, and 1 (< 1%) Native American.

## Results

Data were analyzed using a nonparametric test of significance for ordinal data based on a pretest (i.e., first year of medical school) and posttest (i.e., second year of medical school) methodology for independent samples. A Mann Whitney U test (p<.05) was conducted to determine if changes in attitudes about Team-Based Learning occurred between the first and second year of medical school (See Table 1). Means and standard deviations for individual items on the survey in the categories of Overall Satisfaction with Team Experience, Team Impact on Quality of Learning, Satisfaction with Peer Evaluation, Team Impact on Clinical Reasoning Ability, and Professional Development can be found in Table 1. Aggregate scores for each of the categories are also listed in Table 1. These scores were calculated by averaging the means for the individual items in each category.

**Table 1.** Results of Mann Whitney U Test of Significance (N = 180)

	Year One		Year Two			
Question	M	SD	M	SD	*z*	*p*
**Overall Satisfaction with Team Experience**	4.05	.82	4.10	.74	-1.190	.234
I have found working as part of a team in my classes to be a valuable experience	4.13	.81	4.24	.73	-2.26*	.024
In most of the teams I have been on, the other team members have generally contributed as much as I have	3.94	.99	4.12	.77	-2.30*	.021
In most of the teams I have been on, the team has worked well together	4.21	.76	4.26	.66	-.532	.595
In most of the teams I have been on, I felt the other team members respected me	4.27	.64	4.20	.64	-1.71	.087
I have found teamwork to be a productive use of course time	3.69	.92	3.70	.92	-.275	.784
						
**Team Impact on Quality of Learning**	3.72	.96	3.75	.89	-.364	.716
I have found that teams help me learn course material more than if I just studied alone	3.75	.97	3.85	.88	-1.50	.133
I have learned more in courses where I have been a member of a team	3.63	.95	3.63	.86	-.086	.932
I have found being part of a team improves my course grades	3.79	.97	3.78	.92	-.270	.787
						
**Satisfaction with Peer Evaluation**	3.55	.98	3.38	1.02	-3.653*	<.001
I have found that my peers have been fair in judging my contributions to a team	4.27	.703	4.29	.723	-.735	.462
I have found that peer evaluation motivates me to work harder	3.32	1.10	3.07	1.14	-3.81*	<.001
I have generally liked the use of peer evaluation as part of my team experience	3.18	1.07	3.00	1.09	-2.62*	.009
I have found that peer evaluation motivates me to work more collaboratively	3.43	1.06	3.16	1.11	-4.17*	<.001
						
**Team Impact on Clinical Reasoning Ability**	3.94	.83	3.88	.80	-1.206	.228
I have found that being on a team has helped me become better at problem solving	3.82	.85	3.75	.85	-1.26	208
I have found that teams make good decisions	4.13	.75	4.09	.73	-.945	.344
Being part of a team discussion has improved my ability to think through a problem	3.87	.88	3.81	.83	-1.33	.183
						
**Professional Development**	3.85	.86	3.61	.94	-5.415*	<.001
I have found that working with a team helps me develop skills in working with others	4.23	.75	4.03	.81	-4.61*	<.001
I have found that working with a team has helped me develop cooperative leadership skills	4.09	.77	3.85	.85	-4.97*	<.001
I have found that working with a team has helped me develop more respect for the opinions of others	4.00	.81	3.82	.88	-3.41*	<.001
I have found that working with a team has enhanced my sense of who I am	3.07	1.17	2.75	1.22	-4.33*	<.001

1 = Strongly Disagree 2 = Disagree 3 = Mixed Opininon 4 = Agree 5 = Strongly Agreee

*Significant at .05 level

Overall findings of this study showed that significant changes in attitudes occurred in the areas of Professional Development, Satisfaction with Team Experience, and Satisfaction with Peer Evaluation. Students reported more positive attitudes during the first year of medical school for the areas of Professional Development and Satisfaction with Peer Evaluation. For Satisfaction with Team Experience, more positive attitudes were noted during the second year of medical school. No significant changes in attitudes between the first and second year of medical school were noted for the areas of Team Impact on Quality of Learning and Team Impact on Clinical Reasoning Ability.

**Overall Satisfaction with Team Experience** - A comparison of overall mean scores for statements in this category suggests that during the first year and second year of medical school students’ attitudes about their satisfaction with their team experience were favorable. With regard to how attitudes changed, mean scores on the item about working as part of team being a valuable experience increased from first to second year. The students’ attitudes about their team members contributing as much as they did also improved from their first year of medical school to the second. It is possible that students’ participation in teams during their first year helped them to become more adept at working in teams and become contributing members during their second year. This may have also helped them to find more value in the team experience during year two. No statistically significant changes were noted for students’ attitudes about the team working well together, team members’ respect for them, or their view of teamwork as a productive use of their time.

**Impact on Quality of Learning** - A comparison of overall mean scores for statements in this category suggests that students’ responses to statements about how working in a team impacted their learning fell into the “mixed opinion” range. Items in this category asked if working in a team helped them to learn course material better than if they had studied alone, if their course grades improved because they were part of a team, and if they learned more in courses where they had been a member of a team. No statistically significant changes in students’ attitudes were noted from first to second year in these areas.

**Satisfaction with Peer Evaluation** - A comparison of overall mean scores for statements in this category suggests that students’ responses to statements about peer evaluation fell primarily in the “mixed opinion” range. Students’ attitudes about their satisfaction with peer evaluation tended to decline from the first year of medical school to the second year. Statistically significant declines in students’ attitudes were noted for the role of peer evaluation in motivating a student to work harder and/or more collaboratively, as well as for how well students liked the use of peer evaluation. No statistically significant change was noted in students’ attitudes toward their peers being fair regarding their judgment of students’ contributions to a team.

**Team Impact on Clinical Reasoning Ability** - A comparison of overall mean scores for statements in this category suggests that students’ responses to statements about how working in teams impacted their clinical reasoning ability also fell primarily in the “mixed opinion” range. No statistically significant changes were noted in students’ attitudes in these areas from first to second year. Although there was no change, students in both years agreed that being on a team helped them be a better problem solver, that teams make good decisions, or that being part of a team improved their ability to think through a problem. It is possible that a change in students’ attitudes in these areas would be more likely to occur after students begin their clinical training in their third year of medical school.

**Professional Development** - A comparison of overall mean scores for statements in the Professional Development category suggests that “mixed opinion” responses predominated. Students’ attitudes about their professional development tended to decline from the first year of medical school to the second year. Statistically significant declines in students’ attitudes were noted for items as follows: working on a team enhanced a sense of who they are, working with a team helped them to develop skills in working with others and to develop cooperative leadership skills, and working with a team helped them to develop more of a respect for the opinions of others. These findings may suggest that team-learning activities related to professional development have a stronger impact on students during their first year of medical school and that this benefit is perceived by students to be somewhat less during their second year.

## Conclusions

This study demonstrates that students’ attitudes about working within teams, their sense of professional development, and comfort and satisfaction with peer evaluation change from first to second year in a curriculum using Team-Based Learning. Peer evaluation seems to be more meaningful to students during their first year of medical school than in their second year. Peer evaluation is an area that students have struggled with in Team-Based Learning; and in the past, our sense has been that students felt uncomfortable completing peer evaluations and receiving peer feedback. Due to this, it is possible that the more often students were asked to complete peer evaluations (i.e., multiple times during year 1 and year 2), the less meaningful they were to them. Peer evaluation tools and approaches have evolved since the time of this study as has students’ familiarity with the peer evaluation process. Improving peer evaluation in Team-Based Learning continues to be a hot topic among Team-Based Learning experts. Given that peer evaluation is an integral part of the Team-Based Learning approach, it may be helpful for medical educators to determine how to increase its value for motivating students to work harder or more collaboratively.

Regarding satisfaction with team learning, although not officially hypothesized, we did expect that satisfaction with team experience would increase from year 1 to year 2. Team-Based Learning was a new concept for students and as they (as well as the faculty) became more comfortable and familiar with it as an instructional modality, it makes sense that their satisfaction improved from year 1 to year 2 regarding team members’ contributions and teamwork being seen as a valuable experience.

The decline from year 1 to year 2 in scores in the category of professional development was most interesting for us to ponder. It is possible that Team-Based Learning had more of an impact during the students’ first year because of where students are with their education and learning curve related to working in teams. By second year, students may have become somewhat conditioned to the medical education environment and may feel that advances in their professional development related to Team-Based Learning tapered off or had less of an impact.

Lastly, given the impact on quality of learning, scores were at the higher end of the “mixed opinion” range and remained in that range from year 1 to year 2. The fact that this remained stable is a positive outcome. It would have been concerning had it dropped off from year 1 to year 2.

A limitation of this study is that students’ attitudes were assessed during the years when Team-Based Learning was first integrated into the curriculum. Thus, it is possible that the students’ attitudes were affected by the newness of the instructional approach as well as the challenges for faculty regarding adapting a Team-Based Learning approach to their courses. There were many hurdles to surmount during the initial years of integrating Team-Based Learning into the school's preclinical curriculum, and this could have impacted students’ attitudes.

A next step is to explore the evolution of the Team-Based Learning strategy in our curriculum. We intend to administer the attitudes survey to a new group of first-year students at our medical school and follow them through their second year. The Team-Based Learning approach has now been in place for six years at our medical school, and given that the approach is a relatively stable part of the curriculum and more fully developed with a culture to support it, it would be worthwhile to explore the current students’ attitudes. A follow-up study of this nature would contribute to better understanding of patterns associated with attitudinal change when Team-Based Learning is initially integrated into a curriculum versus when it is a fully developed aspect of the curriculum. Given the students’ overall favorable evaluation of courses in more recent years (students’ satisfaction scores with Team-Based Learning courses have consistently averaged 4.00 or higher on a 5-point Likert-type scale), we anticipate that the students’ attitudes toward a Team-Based Learning approach may have improved; results of a follow-up study will likely evidence fewer responses by students in the “mixed opinion” range. For example, our sense is that the faculty is more experienced and adept in their ability to facilitate the Team-Based Learning curriculum and that students like the peer feedback more now. Further exploration is necessary to confirm our hypothesis.

In closing, as medical schools search for teaching/learning strategies that address the important professional competencies of interpersonal skills, communication, teamwork, and the giving and responding to feedback, Team-Based Learning should continue to be explored as a strategy for promoting the development of these non-cognitive domains.
